# A novel deep learning-based perspective for tooth numbering and caries detection

**DOI:** 10.1007/s00784-024-05566-w

**Published:** 2024-02-27

**Authors:** Baturalp Ayhan, Enes Ayan, Yusuf Bayraktar

**Affiliations:** 1 Department of Restorative Dentistry, Faculty of Dentistry, Kırıkkale University, Kırıkkale, Turkey; 2 Department of Computer Engineering, Faculty of Engineering and Architecture, Kırıkkale University, Kırıkkale, Turkey

**Keywords:** Artificial intelligence, Digital bitewing radiography, Deep learning, Detection, Numbering, Dental caries

## Abstract

**Objectives:**

The aim of this study was automatically detecting and numbering teeth in digital bitewing radiographs obtained from patients, and evaluating the diagnostic efficiency of decayed teeth in real time, using deep learning algorithms.

**Methods:**

The dataset consisted of 1170 anonymized digital bitewing radiographs randomly obtained from faculty archives. After image evaluation and labeling process, the dataset was split into training and test datasets. This study proposed an end-to-end pipeline architecture consisting of three stages for matching tooth numbers and caries lesions to enhance treatment outcomes and prevent potential issues. Initially, a pre-trained convolutional neural network (CNN) utilized to determine the side of the bitewing images. Then, an improved CNN model YOLOv7 was proposed for tooth numbering and caries detection. In the final stage, our developed algorithm assessed which teeth have caries by comparing the numbered teeth with the detected caries, using the intersection over union value for the matching process.

**Results:**

According to test results, the recall, precision, and F1-score values were 0.994, 0.987 and 0.99 for teeth detection, 0.974, 0.985 and 0.979 for teeth numbering, and 0.833, 0.866 and 0.822 for caries detection, respectively. For teeth numbering and caries detection matching performance; the accuracy, recall, specificity, precision and F1—Score values were 0.934, 0.834, 0.961, 0.851 and 0.842, respectively.

**Conclusions:**

The proposed model exhibited good achievement, highlighting the potential use of CNNs for tooth detection, numbering, and caries detection, concurrently.

**Clinical significance:**

CNNs can provide valuable support to clinicians by automating the detection and numbering of teeth, as well as the detection of caries on bitewing radiographs. By enhancing overall performance, these algorithms have the capacity to efficiently save time and play a significant role in the assessment process.

## Introduction

Dental caries is a preventable, chronic, multifactorial, infectious disease that is common in many populations worldwide [1]. Carious lesions, which usually progress slowly, can be a serious health problem that can lead to pain, infection and tooth loss if not treated in early stages [2]. While the perspective on caries treatment was more invasive in the past, today in modern dentistry, this perspective has been replaced by early diagnosis with preventive and minimally invasive treatment methods [3]. Early and accurate detection of caries lesions is a very important step that guides clinical planning by determining the treatment to be applied. Therefore, timely and accurate diagnosis has a key role in a successful treatment [4]. Although there are different methods in the diagnosis of caries lesions, the visual-tactile examination method which is insufficient in the diagnosis of early approximal caries detection is one of the most basic of them [5, 6]. In addition to the visual-tactile examination method, radiological examination which is commonly employed in intraoral imaging plays a crucial role in dental practice. In this regard, panoramic, periapical and bitewing radiography are regularly utilized in practice. Visualization and evaluation of dental status on radiographs are one of the most important steps in disease diagnosis [7, 8]. Today, with the help of digital bitewing radiographs, which have high recall and specificity in the detection of approximal caries, the detection of caries lesions is done easily, cheaply and successfully compared to many methods [9, 10].

As can be understood from all these, radiological imaging and visual-tactile examination methods are essential for detecting carious lesions. In some situations, depending on the experience and attention of the dentist, misdiagnosis or insufficient diagnosis can be made in bustling clinics. The use of computer aided systems can help to dentists during diagnostic process. Also, it can provide the physician to achieve success by assisting with treatment planning and implementation [11, 12].

Artificial intelligence term (AI), which has become very popular in our lives and affects many industries and promises to be a real turning point [13]. AI is a general term that refers to systems, machines or computers that mimic human behaviors and intelligence [14]. It includes different areas of research fields and algorithms. Deep learning (DL) is one of these research areas that has gained popularity in recent years [15]. DL algorithms consist of various artificial neural networks according to the different types of problems such as natural language processing, large language models, computer vision [16]. Convolutional neural networks (CNNs) are supervised deep learning algorithms that are artificial neural networks inspired by the mammalian visual system [17]. CNNs are actively used in many computer vision problems in dentistry and medicine for improving health services and increasing efficiency [13, 18].

The adoption of CNNs in the diagnosis of caries in dentistry is a successful method as can be understood from the studies carried out. There are many studies on CNN and similar neural networks [19–24]. Bitewing radiographs are often used by dentists as an assistant tool to diagnose dental caries [25]. In some of the researches, caries diagnosis studies were performed on bitewing radiographs [19, 20, 26, 27]. Correct detection and numbering of teeth during the examination can reduced the examination time and provide a better diagnosis [28]. Yasa et al. [29] researched teeth detection and numbering in bitewing radiographs. Also, there are similar studies have been searched with in panoramic or bitewing radiographs [30–34]. These studies showed that the developments based on DL in the health industry can be an assistant and a second opinion provider in the daily clinical practice of the physician [35]. To the best of our knowledge, no study has been investigated that teeth detection, numbering, and caries detection at the same time in digital bitewing radiographs utilizing a CNN approach based on DL. The proposed method makes this study unique compared to existing literature.

The aim of this study was to automatically detecting and numbering teeth in digital bitewing radiographs obtained from patients, and to evaluate the diagnostic efficiency of decayed teeth in real time, with a pipeline architecture using CNNs. Thus, it is planned to reduce the chair time, to assist the physician and to increase the effectiveness of the treatment by making the diagnosis more effective during the examination.

A successful proposal introduces a novel DL-based pipeline architecture for teeth numbering and caries detection. The architecture enhances the preservation of crucial information in bitewing images by incorporating convolutional block attention modules (CBAMs) and average pooling layers into the YOLOv7 model. This implementation results in a more efficient performance in teeth numbering and decay detection compared to the original YOLOv7 model. Additionally, an algorithm has been developed to assess teeth with caries by correlating the numbered teeth with the detected caries.

## Material and methods

### Sample size determination

The determination of the sample size for the test dataset was performed with Fisher’s exact test A design incorporating clustering was employed because of every digital bitewing radiograph contained averagely 16 surfaces which was calculated by design effect (DE). Design effect (DE), which assesses the performance of a particular sampling method compared to that of a simple random sample, is a valuable tool in considering the precision of a cluster sample. Equation of DE is 1 + (m-1) x ICC, in where m represents size of cluster, and ICC is the intraclass correlation coefficient. ICC of 0.2 was assumed based on Masood et al. [36]. According to previous study’s power analysis, required test set units multiplied with DE [37]. When DE = 1 + (16 – 1) × 0.2 = 4, the total surfaces required should be 2136 or 134 bitewing radiographs. In the present study, test dataset contained 170 bitewing radiographs.

### Image dataset

A total of 1170 digital bitewing radiographs of adult patients aged over 18 were collected from radiology archive of Kırıkkale University, Faculty of Dentistry for this study. Bitewing radiographs which obtained from patients who came for examination and/or treatment between January-2018 and January-2023 included by examining. No further details, such as gender, age or acquisition date of images was revealed, since the radiographs were collected anonymously. In the study, bitewing radiographs containing patient positioning errors, technical errors, fixed prosthetic restoration and fixed orthodontic braces that would complicate or completely prevent the diagnosis did not include in the dataset. Approval for this study was obtained from the non-interventional Clinical Research Ethical Committee of Kırıkkale University. (Decision Number: 2022.11.14) The study adhered to the ethical guidelines stated in the Declaration of Helsinki.

**Fig. 1:**
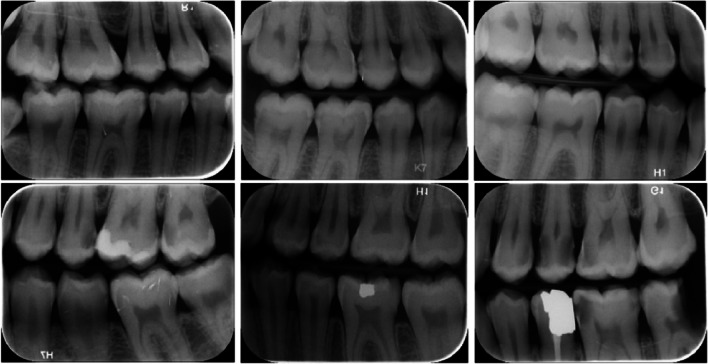
Image samples from the dataset

All digital bitewing radiographs were taken by a Gendex Expert DC (Gendex Dental Systems, IL, USA) device that is located in the clinics with a focal spot value of 0.04 mm, tube voltage 65 kVp, tube current 7 mA. Exposure time, distance, and quality of radiographs can be variable, because of the digital bitewing radiographs were captured by various personnel across different departments. Each digital bitewing radiograph was anonymized and saved as.jpg format. Maxillary and mandibulary dental arches were distinctly visible on digital bitewing images. The assessment included all visible teeth, excluding restorations present on the proximal surfaces of the teeth. Because of the aim of this study was evaluating the proposed model performance for detecting primary approximal caries and numbering teeth. The collected digital bitewing radiographs displayed heterogeneous resolutions in terms of their sizes. For this reason, all resolutions were adjusted to 640 × 480 pixels, such as a previous study [19]. After this process, the dataset was randomly split as follows: 85% for training and validation (1000 bitewings) and 15% for testing (170 bitewings). Thus, 1000 digital bitewing radiographs that obtained with random distribution were used for training the proposed CNN model. The remaining 170 digital bitewing radiographs were used for evaluating the model. In the training dataset, 9344 teeth were numbered. There are 16674 approximal surfaces and 2289 of them are caries. The lesion prevalence of the training data is 24%. Details of the dataset are given in Table 1 and image samples from the dataset are given in Fig. 1.

**Table 1:** Summary of whole dataset

	Train	Test
Decay	2289	602
Numbering	9344	1679
Total surfaces	16,674	2842
Lesion prevalence	24%	21.16%
Total image	1000	170

### Study design

**Fig. 2:**
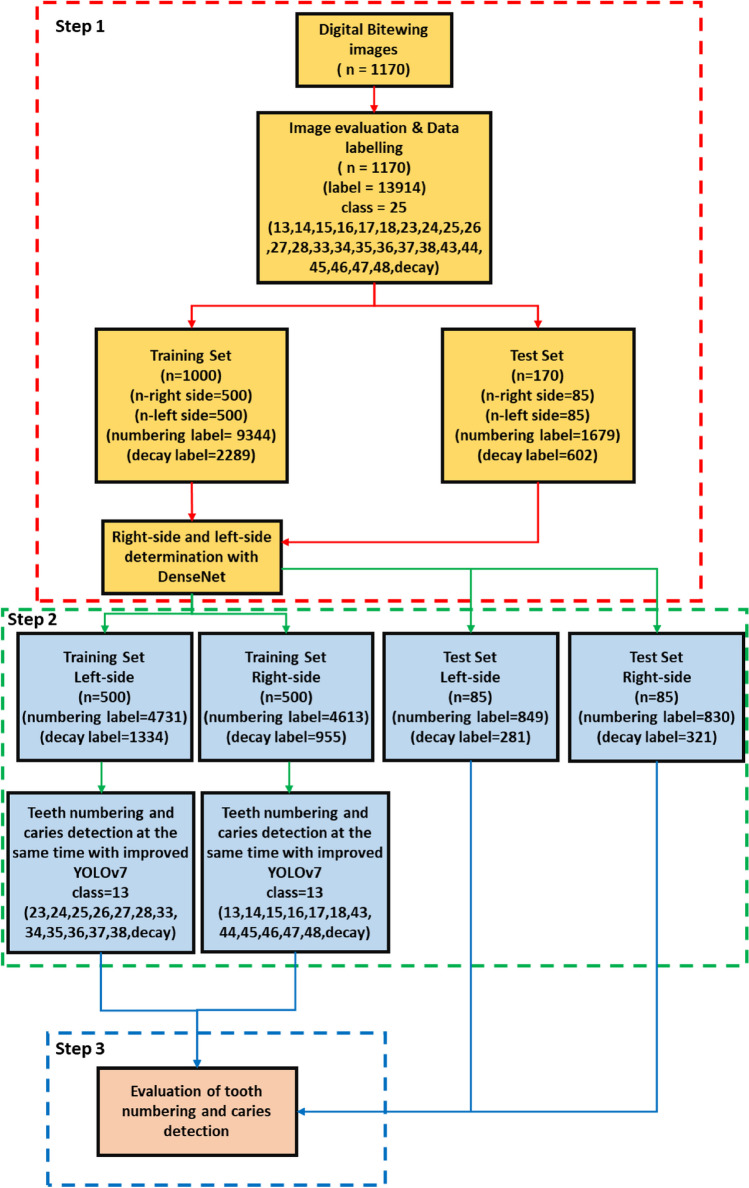
Diagram of the implementation of the proposed architecture

In this study, a pipeline architecture that can simultaneously detect teeth numbering and caries in bitewing images is proposed. The proposed architecture consists of 3 basic steps. A diagram illustrating the application of the proposed architecture is given in Fig. 2.

### Image evaluation and data labeling

**Fig. 3:**
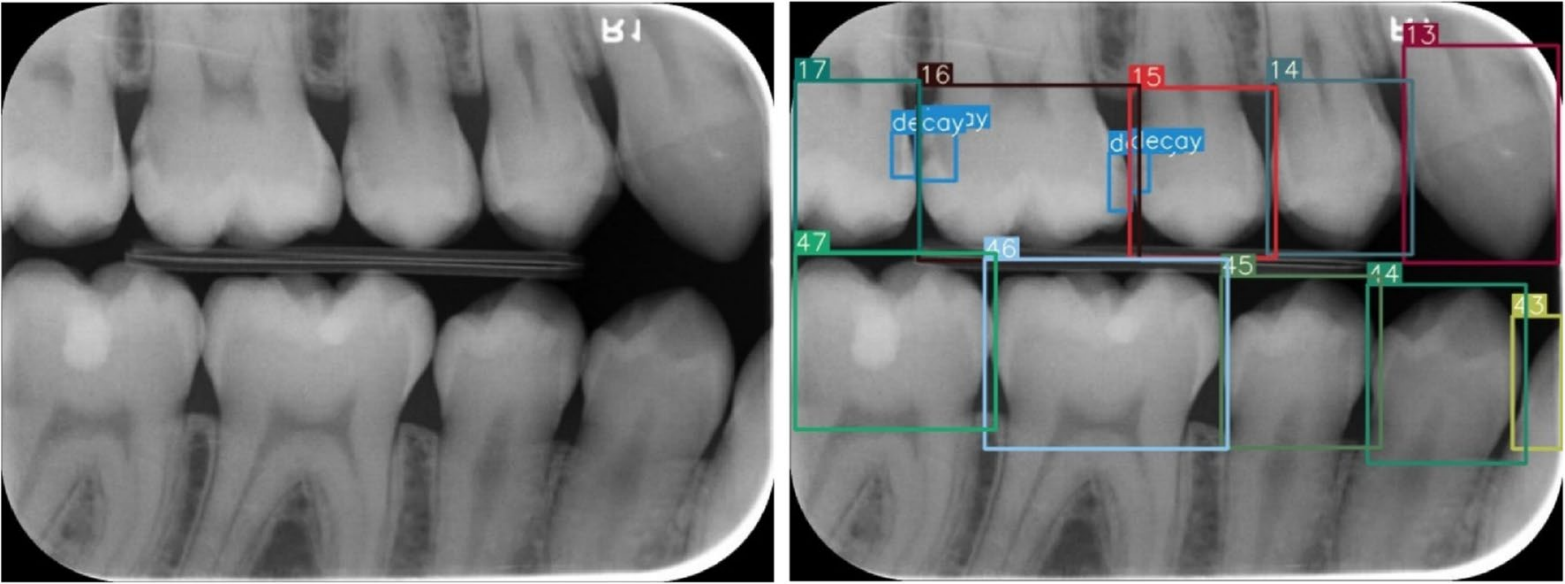
Image samples from the labeling process

A restorative specialist (author 3) with 13 years of experience and a research assistant (author 1) with 4 years of experience generated ground truth annotations for the images using software program which developed in Python programming language. For that, annotators labeled bounding boxes around all teeth while simultaneously providing a class label for each box with the tooth number according to Federation Dentaire International (FDI) tooth notation system (16–17-18–26-27–28- 36–37-38–46-47–48 (molars), 14–15-24–25-34–35-44–45 (premolars) and 13–23-33–43 (canines)). Also, caries lesions labeled as decay in digital bitewing images. While in the data labeling procedure, annotators reviewed the bitewing images at the same time and as a result of the agreement, labeling was processed (Fig. 3).

After this part, approximal caries lesions on the tooth surfaces have been labeled with bounding boxes by the annotators with the same agreement procedure. The software that used for labeling was automatically converted the box information and labels according to the needs for training of YOLO.

### Detecting bitewing image’s side via convolutional neural network

The first step of the proposed pipeline architecture was to automatically detect whether the bitewing images belong to the left or right sides. This step was very important for the numbering step of the teeth. Because the success rate of the numbering process was found to be low in the first training without separating the right and left sides. For this purpose, a DenseNet CNN architecture was utilized, which was introduced in 2016 by Huang et al. [38]. DenseNet architecture uses dense connections, which connect each layer to other layers in a forward manner. Dense connections have benefits, such as reducing model parameters, decreasing vanishing-gradient problem, encouraging feature reuse, and strengthening feature propagation along layers. Four DenseNet architectures exist in the literature (DenseNet-121, DenseNet-169, DenseNet-201, DenseNet-264). In this study, a pre-trained DenseNet-121 architecture on the ImageNet dataset was utilized and was re-trained using transfer learning and finetuning methods to detect whether the images belong to the left or right sides. The main reason for selecting the DenseNet-121 is that it is more lightweight than other architectures, so it can be trained faster. The fine-tuned DenseNet-121 model has 7,564,865 parameters, one fully connected layer with 512 neurons and one neuron output with sigmoid activation function. The proposed model trained for 30 epochs with 32 batch size, 0.001 initial learning rate. Binary cross entropy was used as the error function and Adam algorithm was preferred for the optimizer.

In training process, 1000 bitewing images, 500 left and 500 right, were used. 10% of the training data was used for validation. 170 bitewing images (85 left and 85 right) were used to evaluate classification performance of the model. According to the test result, CNN model was achieved %100 accuracy on the test dataset. A diagram summarizing this phase is given in Fig. 2.

### Improved YOLOv7 architecture, teeth numbering and caries detection

#### The architecture of original YOLOv7

The second stage of the study was teeth numbering and caries detection from the bitewing images. In this step, DL-based object detection algorithms were taken into consideration due to the nature of the problem to be solved. There exist two types of DL-based object detection algorithms: region-based (two stage) and regression-based (one stage). In region-based algorithms, firstly, candidate regions containing possible objects in identifies, then these regions are classified to determine object types in them. Fast-RCNN [39], Faster-RCNN [40], Mask-RCNN [41] algorithms are some examples of this category. On the other hand, one-stage algorithms detect and classify objects at the same time. The algorithms in this category are YOLO (You Only Look Once) and its different versions such as YOLO9000, YOLOv3, YOLOv4, YOLOv5, YOLOv6, YOLOv7 [42, 43]. One-stage algorithms offer a faster detection speed than two-stage ones, this advantage makes them popular in object detection problems.

**Fig. 4:**
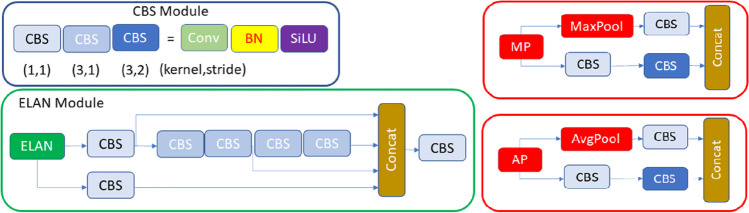
CBS, ELAN, and MP modules representation

**Fig. 5:**
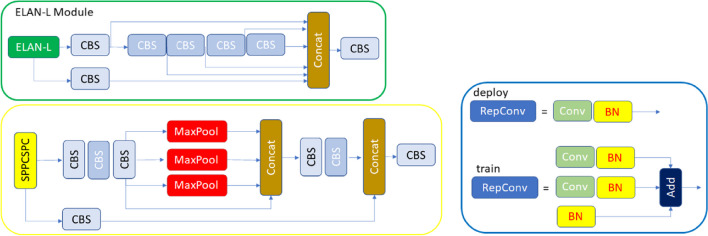
ELAN-L, Rep and SPPCSPC modules representation

In YOLOv7, one of the latest versions of the YOLO series, some improvements have been made in the loss function, backbone network and activation function. In this way, the model has achieved improved detection accuracy and accelerated detection speed. For this reason, the YOLOv7 algorithm was chosen to be used in the study. The original YOLOv7 architecture consists of four modules: input, backbone, neck and head. The input module resizes the input image to a standardized pixel size (640 × 640) to fulfill the input size specifications of the backbone module. The backbone of the original architecture consists of convolutional blocks, batch normalization process, and SiLU activation function (CBS), extended efficient long-range attention network (E-ELAN) and MaxPool-1 (MP1) modules. The task of the backbone part is to extract feature maps from the input image. The CBS module consists of three components: convolution blocks, batch normalization process (BN), and SiLU activation function (sigmoid-weighted linear unit activation function) (Fig. 5). The ELAN module comprises multiple CBS modules that ensure the input and output feature sizes remain unchanged. By guiding the computational blocks of different feature groups to learn various features, the network's learning capacity is enhanced while preserving the integrity of the original gradient path (Fig. 5). The MP1 module is divided into two branches specifically an upper branch and a lower branch and consisting of CBS modules and the max pooling (MaxPool) layer (Fig. 4). In this module, the upper branch utilizes MaxPool to capture the maximum value information from small local areas, while the CBS modules in the lower branch extract all valuable information from small local areas. This dual approach enhances the network's feature extraction capability.

The neck module consists of ELAN-L, Upsample, MP2 and SPPCSPC modules using the PAFPN Path Aggregation Feature Pyramid Network (PAFPN) architecture. The neck part combines the feature maps obtained from the different layers of the backbone module and sends them as input to the head module. The SPPCSPC module consists of CBS modules and MaxPool layers with different sizes of parallel max pooling (Fig. 5). The ELAN-L module has a similar structure to the ELAN module, but it combines five different outputs (Fig. 5). The Up-sample module consists of the CBS module and is used for upsampling. The Head module includes of a Rep block to set the number of image channels for the features output from the neck part. This is followed by the application of 1 × 1 convolution module, which predicts the category, confidence, and bounding box. The REP module is inspired by RepVGG [44] and employs a simplified convolution process during the testing phase (Fig. 5). This simplification effectively reduces network complexity without compromising performance.

### Improved YOLOv7 with convolutional block attention module

**Fig. 6:**
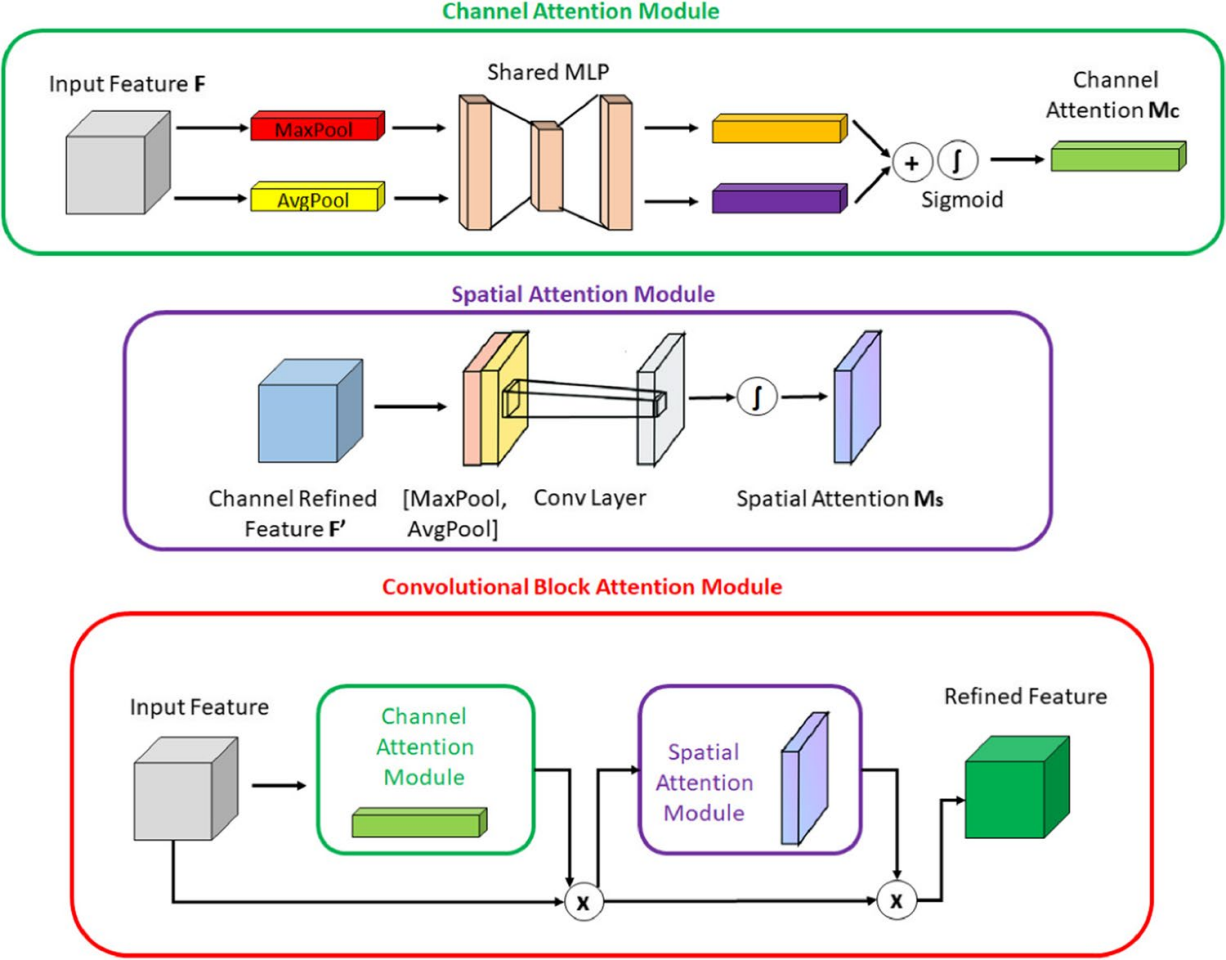
CBAM, CAM and SAM modules representation

The scientific community widely acknowledges the significant role of attentional mechanisms in human vision. A fundamental characteristic of the human visual system is its ability to selectively focus on salient aspects of a scene rather than processing the entire visual field simultaneously. This selective attention enables enhanced capture of visual details [45]. In recent years, researchers have developed various selective attention mechanisms for neural networks. These mechanisms enable neural networks to learn "where" or "what" to focus more on, using explicit dependencies between channels and weighted spatial regions. In this context, the convolutional block attention module (CBAM) was introduced by Woo et al. [46] as an efficient attention mechanism designed for CNNs. Within this context, Woo et al. [46] proposed the convolutional block attention module (CBAM), which is an effective attention mechanism for CNNs. The CBAM module includes of a channel attention module (CAM) and a spatial attention module (SAM). The CAM is focused on the relevant aspects of an input image by exploiting the inter-channel relationships of features. Two spatial context descriptors are generated in the module using two separate pooling methods (average pooling and max pooling). Average pooling (AP) is mainly used to gather spatial information, whereas max pooling retains significantly more contextual details pertaining to the object within the image, thereby enabling more precise channel attention. These generated context descriptors are forwarded to a shared multi-layer network. After the vector obtained from the multilayer network is collected, the channel attention map is generated by applying the sigmoid function. Within the CBAM module, the spatial attention module (SAM) specifically targets the localization of the most informative regions within the input image. Spatial attention computation within the SAM module involves the application of average pooling and max pooling techniques along the channel axis of the input image. The obtained two-channel image is subjected to convolutional processing to convert it into a single-channel feature map, which is then merged and forwarded to a standard convolutional layer to create an efficient feature descriptor. The output from the convolutional layer is passed through a sigmoid activation layer. The Sigmoid function, which is a probability-based activation, transforms all values to a range between 0 and 1, thereby revealing the spatial attention map. In summary, CBAM combines the two attention modules described above to determine what is important in the input image and to locate the descriptive regions within the image. Figures of CBAM, CAM and SAM modules are given in Fig. 6.

When reviewing the studies in the literature, it is seen that there are successful combinations of the CBAM module in CNNs in the solution of computer vision problems [47, 48]. However, creating a neural network is a complex process that requires consideration of the utilized dataset, the selection of optimized metrics, and the availability of computational resources.

**Fig. 7:**
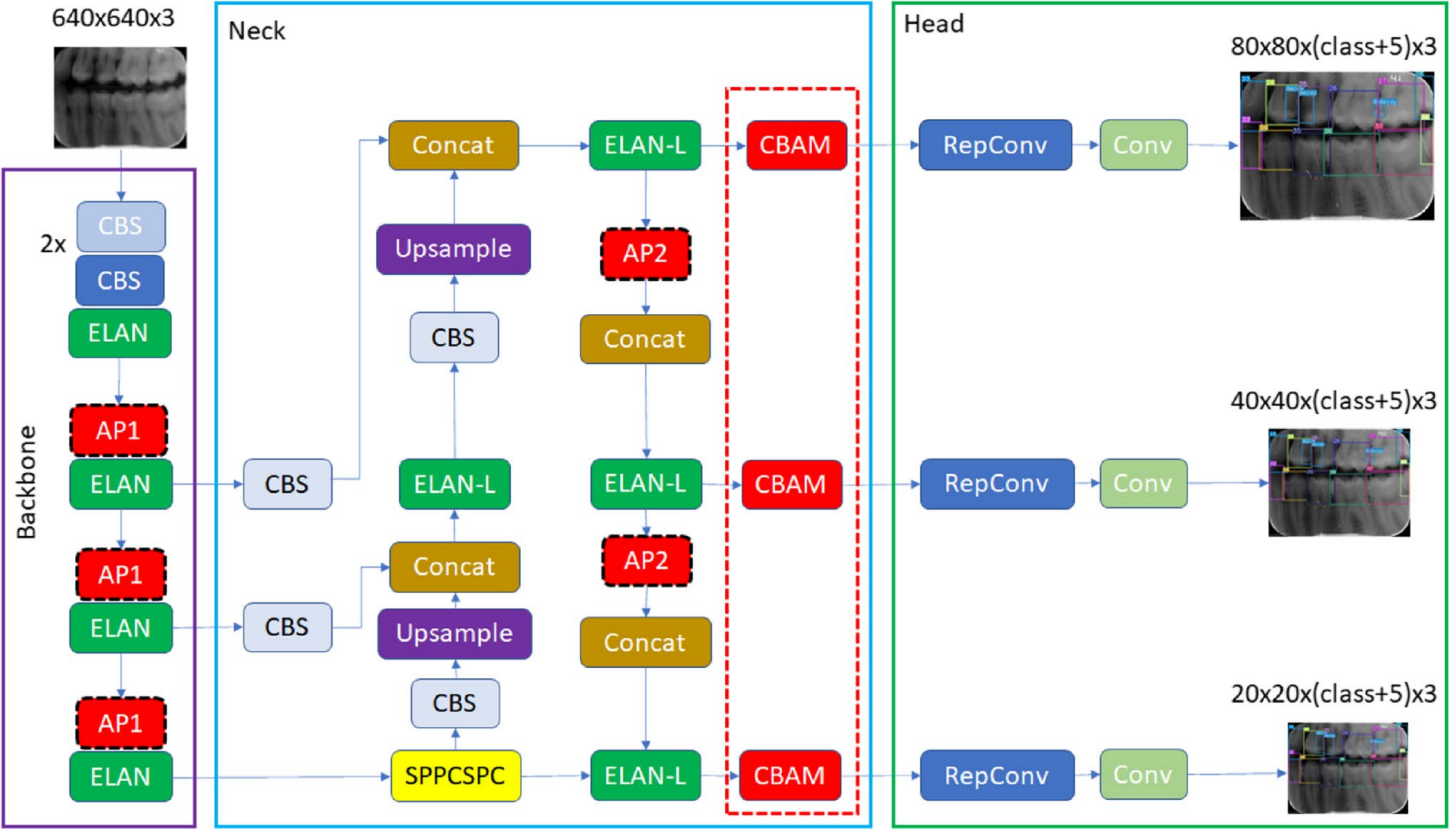
Improved YOLOv7 with CBAM, and AP modules

In this study, we injected CBAM layers in the YOLOv7 model as a result of our experience with CNNs and various tests we performed. After conducting various experiments, three CBAM modules have been placed at three specific locations where the intersections of the neck and head in the YOLOv7 architecture. The selection of the number of integrated CBAM blocks during the incorporation of CBAM modules was done meticulously to minimize any significant increase in the computational overhead on the network. The integration of CBAM modules into the YOLOv7 architecture by researchers to develop novel designs for solving various problems has emerged as a highly popular research area [49, 50]. Therefore, the determination of position, number of blocks, and their integration with other network components are design decisions that can vary depending on the specific problem under consideration. To the best of our knowledge, there is currently no known study that specifically addresses the region we have incorporated in our research. Figure 7 provides an illustration of the improved model and details about the location where CBAM modules are positioned.

Another improvement we have made in our proposed YOLOv7 model is to use average pooling (AP) instead of max pooling (MP) in subsampling layers. In the original YOLOv7, subsampling is done at four different locations. In these subsamples, the average pooling method was used instead of the max pooling. Max pooling returns the maximum value in each window, while average pooling returns the average value in each window. The benefit of using average pooling instead of max pooling is that it can help reduce overfitting. Max pooling is more likely to memorize as it preserves only the strongest feature in each window that may not represent the entire window. On the other hand, average pooling considers all features in each window, which can help preserve more information. Additionally, average pooling can help smooth out the output, which can be useful when the input is noisy or volatile. In this context, the changes we made are given in Fig. 7. It has been observed that this improvement has a positive effect on detection performance.

The architecture of YOLOv7-AP-CBAM obtained with the updates made in the YOLOv7 architecture was used in the numbering of teeth and in the detection of caries. In this context, two different models were trained for the right and left parts of the mouth. Since the numbering performance of a single model remained low in the tests, two different model training methods were used. The models for both sides were trained with the following hyperparameters: batch size 16, epochs 20, input image size (640,640), initial learning rate 0.01, optimizer SGD algorithm, momentum 0.937, weight decay 0.005. A flowchart summarizing this stage is given in Fig. 7.

### Evaluation of teeth numbering and caries detection matching

**Fig. 8:**
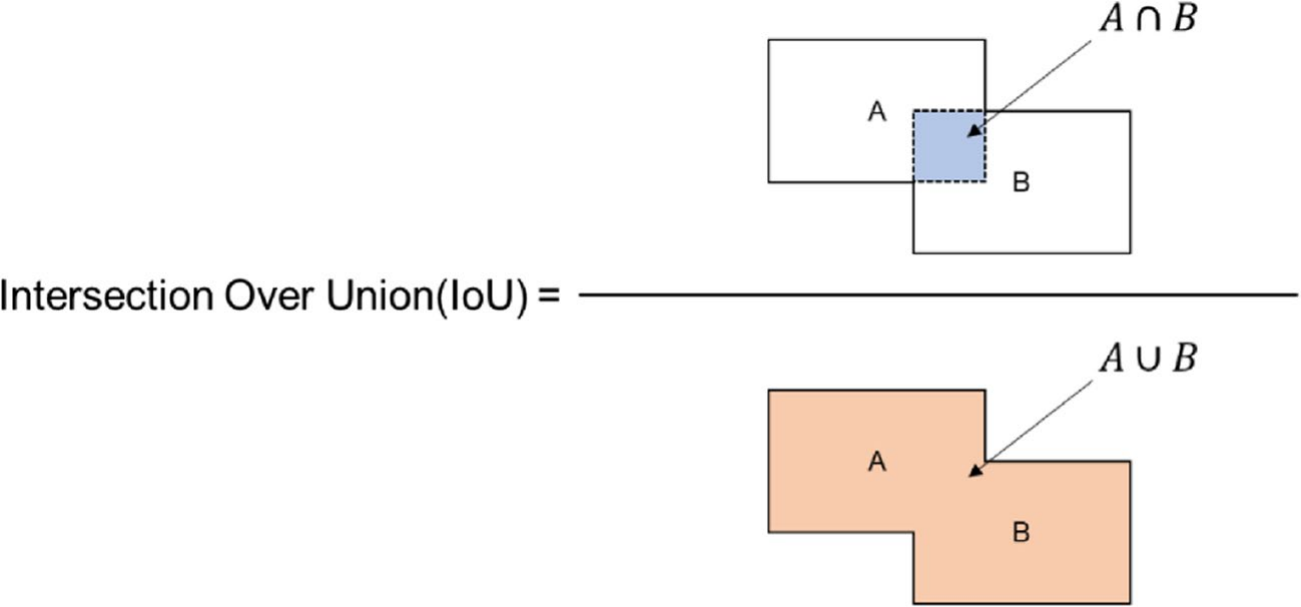
The formula of IoU. A represents the numbering box, while B represents the caries box

In the last stage of the proposed architecture, it was aimed to give the output of the system which tooth had caries by matching the numbered teeth and the detected caries. It has been determined whether there was a bounding box drawn for caries in the bounding boxes drawn for tooth numbering. If there was caries in the numbered bounding box, it was reported which tooth had caries. The Intersection Over Union (IoU) value was used to detect caries in the numbered bounding box (see Fig. 8).

**Fig. 9:**
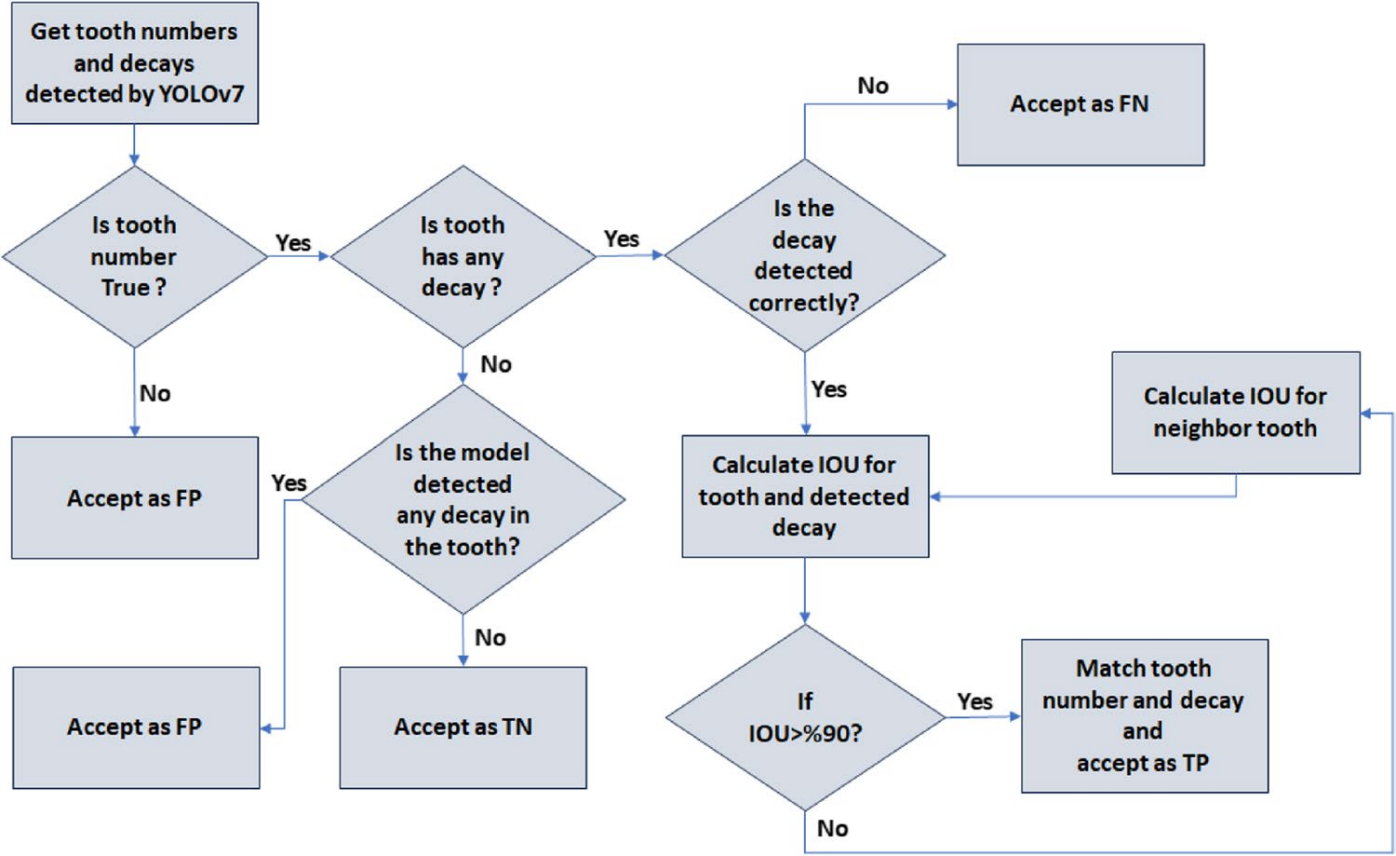
Diagram of teeth numbering and caries detection matching decision

If the IoU value was above 90%, it was accepted that the number of the tooth with caries label was determined correctly. The flow chart of the algorithm we developed for this purpose is given in Fig. 9.

### Experimental setup

All experiments in this study are executed on NVIDIA 1080 Ti GPU with 12G video memory size and two Intel Xenon CPU processors with 64 GB ram on the Ubuntu operating system. The experimental running environment includes Python programing language, Pytorch [51], Keras [52] DL frameworks, and OpenCV computer vision library [53].

### Performance evaluation analysis

To evaluate the success of the proposed model, a confusion matrix, that summarizes the actual and predicted situations in a table, was used together with the following procedures and metrics. The confusion matrix that we adapted for evaluating performance of teeth numbering and caries detection matching is given below. Also, the performances of teeth detection, numbering, and caries detection were evaluated individually.

**Table:** 

Adaptation of the confusion matrix for evaluating performance of teeth numbering and caries detection matching
TP: the output in which the model correctly predicts the positive class (teeth correctly detected, correctly numbered, caries correctly detected on bitewing radiographs)
FP: the output in which the model incorrectly predicts the positive class (teeth correctly detected, correctly numbered, but caries prediction on sound surface; teeth correctly detected, but regardless of caries incorrectly numbered)
FN: the output in which the model incorrectly predicts the negative class (teeth correctly detected, correctly numbered, but no caries prediction on decayed surface)
TN: the output in which the model correctly predicts the negative class (teeth correctly detected, correctly numbered, no caries prediction when there was no caries)

Initially, True Positive (TP), False Positive (FP), False Negative (FN) and True Negative (TN) rates were determined. Then, Accuracy (Eq. 1), Recall (Eq. 2), Specificity (Eq. 3), Precision (Eq. 4), F1 – Score (Eq. 5) and IoU (Eq. 6) scores were calculated. IoU is defined as the measure of overlap between the actual bounding box labeled by the annotator for an object and the bounding box predicted by the model. By comparing the IoU value with the threshold value determined prior to model training, it is possible to interpret the success of the trained model in object detection. The F1 score, which is considered the harmonic average of precision and recall, is commonly used for providing a balanced evaluation of a classification model's performance.

The equations are shown below (Eqs. 1–6).

1 $$Accuracy =\frac{TP+TN}{TP+FP+FN+TN}$$

2 $$Recall=\frac{TP}{TP+FN}$$

3 $$Specificity=\frac{TN}{TN+FP}$$

4 $$Precision=\frac{TP}{TP+FP}$$

5 $$F1-Score=2*\frac{Precision* Recall}{Precision+Recall}$$

6 $$IoU= \left|\frac{A \cap B}{A \cup B}\right|$$

## Results

In the testing dataset which include 170 digital bitewing radiographs, there were 1679 teeth, 2842 approximal surfaces, 1679 numbering labels, 602 caries labels. The lesion prevalence of the testing data is 21.18%.

Without splitting into right and left sides, the Recall, precision, and F1-score values were 0.409, 0.236, and 0.299 for teeth numbering, and 0.676, 0.707, and 0.691 for caries detection, respectively (Table 5).

After splitting into right and left sides, the Recall, precision, and F1-score values were 0.994, 0.987 and 0.99 for teeth detection, 0.974, 0.985 and 0.979 for teeth numbering, and 0.833, 0.866 and 0.822 for caries detection, respectively (Table 2 and 3).

**Table 2:** Evaluation metrics of the teeth detection model

	Recall	Precision	F1-score
Left side	0.995	0.987	0.99
Right side	0.993	0.988	0.99
All	0.994	0.987	0.99

**Table 3:** Evaluation metrics of the teeth numbering and caries detection model

	Recall	Precision	F1—score
Left side teeth numbering	0.976	0.978	0.977
Right side teeth numbering	0.972	0.993	0.982
Left side caries detection	0.857	0.833	0.844
Right side caries detection	0.810	0.900	0.800
Left side—numbering and caries detection	0.966	0.966	0.966
Right side—numbering and caries detection	0.959	0.985	0.968
Left and right—side numbering and caries detection—All	0.962	0.975	0.967
Teeth numbering—All	0.974	0.985	0.979
Caries detection—All	0.833	0.866	0.849

For teeth numbering and caries detection matching performance; the Accuracy, recall, specificity, precision and F1—Score values were 0.934, 0.834, 0.961, 0.851 and 0.842, respectively (Table 4).

**Table 4:** Evaluation metrics of the teeth numbering and caries detection matching model

	TP	FP	FN	TN	Accuracy	Recall	Specificity	Precision	F1-score
Left side	240	48	47	1102	0.933	0.836	0.958	0.833	0.834
Right side	260	30	66	1049	0.931	0.797	0.972	0.896	0.843
All	500	78	113	2151	0.932	0.815	0.965	0.865	0.839

**Fig. 10:**
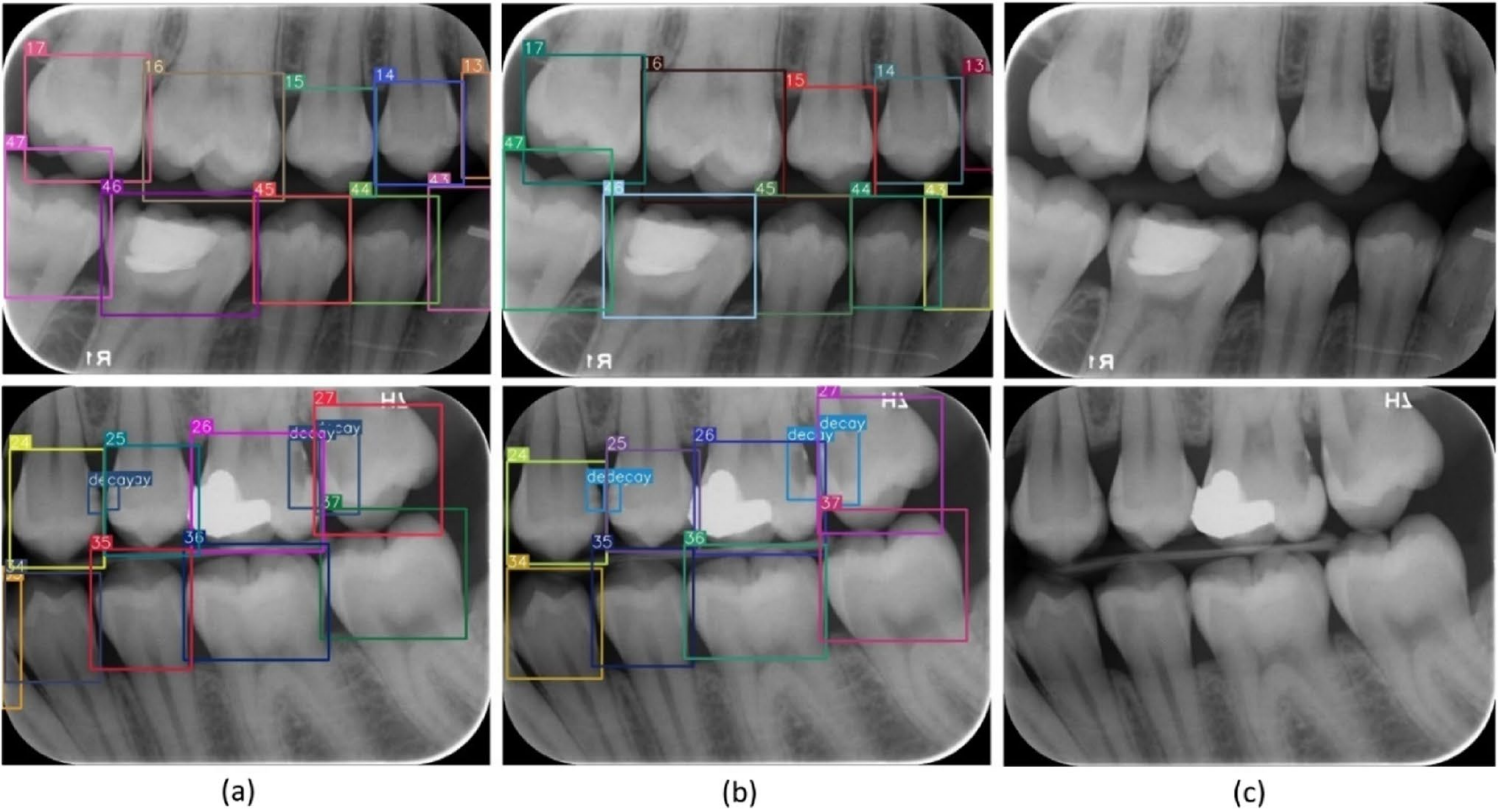
Image samples for detecting and numbering teeth, as well as detecting caries with the proposed YOLOv7-AP-CBAM model on digital bitewing radiographs. **a** labels of improved YOLOv7-AP-CBAM model, **b** labels of ground truth, **c** raw image

The improved YOLOv7-AP-CBAM models performed well in detecting and numbering teeth, as well as detecting caries at the same time on digital bitewing radiographs (Fig. 10).

### Ablation Experiments

This study performed ablation experiments to evaluate the impact of various enhancements on the model's performance. Initially, the original YOLOv7 model was employed to extract features without splitting into right and left sides. Subsequently, the original YOLOv7 model was modified to include the split into right and left sides, as well as the integration of AP and MP with CBAM. Finally, AP with CBAM was applied. The teeth numbering and caries detection recall, precision, and F1-score of the model with used AP and CBAM modules significantly improved compared to initial of this study. The experimental results are presented in Table 5.

**Table 5:** Ablation comparison of performance enhancements on the model

	Recall		Precision		F1-score	
Original YOLOv7-MP without splitting into right and left	Decay all	0.676	Decay all	0.707	Decay all	0.691
	Numbering all	0.409	Numbering all	0.236	Numbering all	0.299
Original YOLOv7-MP with splitting into right and left	Right side decay	0.659	Right side decay	0.803		
	Left side decay	0.696	Left side decay	0.848		
	Decay all	0.677	Decay all	0.825	Decay all	0.743
	Right side numbering	0.971	Right side numbering	0.974		
	Left side numbering	0.979	Left side numbering	0.978		
	Numbering all	0.975	Numbering all	0.976	Numbering all	0.975
YOLOv7-AP	Right side decay	0.626	Right side decay	0.773		
	Left side decay	0.797	Left side decay	0.775		
	Decay all	0.711	Decay all	0.774	Decay all	0.741
	Right side numbering	0.967	Right side numbering	0.977		
	Left side numbering	0.981	Left side numbering	0.983		
	Numbering all	0.974	Numbering all	0.980	Numbering all	0.976
YOLOv7-MP-CBAM	Right side decay	0.579	Right side decay	0.838		
	Left side decay	0.754	Left side decay	0.832		
	Decay all	0.666	Decay all	0.836	Decay all	0.741
	Right side numbering	0.974	Right side numbering	0.983		
	Left side numbering	0.981	Left side numbering	0.978		
	Numbering all	0.977	Numbering all	0.980	Numbering all	0.978
YOLOv7-AP-CBAM (ours)	Right side decay	0.810	Right side decay	0.900	All decay	0.849
	Left side decay	0.857	Left side decay	0.833		
	All decay	0.833	All decay	0.866		
	Right side numbering	0.972	Right side numbering	0.993		
	Left side numbering	0.976	Left side numbering	0.978		
	Numbering all	0.974	Numbering all	0.985	Numbering all	0.979

In CNNs, complexity is described by the total number of parameters of the model and the number of Giga Floating Point Operations (GFLOPs) [54]. We compared basic version of YOLOv7 with improved version of YOLOv7-AP-CBAM. The original YOLOv7 has 314 layers 36,611,228 million parameters, 103.7 GFLOPS, 75.1 MB size in memory. On the other hand, the proposed YOLOv7-AP-CBAM has 350 layers 36,655,485 million parameters 103.8 GFLOPS and 75.2 MB size in memory. A satisfactory performance increase was achieved with a small increase in complexity in the model.

## Discussion

In dentistry, achieving an accurate and precise diagnosis of dental caries is essential for the primary step towards successful treatment implementation. Dental caries diagnosis methods aim to improve the diagnostic process by facilitating the early detection of caries or enabling its objective identification [55]. Although tooth detection and numbering may seem straightforward, the accuracy of tooth numbering and caries detection can be affected by the limited and diverse knowledge of novice clinicians, potentially resulting in inconsistent diagnoses and inadequate treatment approaches. In addition to experience, practitioners' inattentiveness or demanding workloads can also have an impact on the diagnostic results. To prevent all these challenges and facilitate the diagnostic process, a computer-aided system based on DL methods can be employed. DL has gained significant popularity in the medical field due to its outstanding problem-solving capabilities. Within this context, numerous DL architectures have been developed, with especially CNNs standing out for their impressive performance in image recognition tasks [56]. By utilizing CNN models, practitioners can effectively enhance work efficiency and achieve precise outcomes in the accurate diagnostic process [34, 57].

In the current study, the efficiency of the improved CNN model YOLOv7-AP-CBAM is presented for automatically detecting and numbering teeth, as well as detecting caries in digital bitewing radiographs, concurrently. So far, various studies have been conducted in the field of dentistry, utilizing CNNs for the detection, classification, and segmentation of various entities such as objects, tooth, caries, numbering, root fracture, periapical lesions and periodontal bone loss [34, 58–63]. According to these studies, CNN-based systems showed promising results in overcoming diagnosing difficulties and assisting practitioners.

Various dental imaging systems have been used for these studies. Digital bitewing radiography technique is widely employed due to its practicality and advantages. Less radiation doses requirement, time efficiency, image storing, simplicity of image enhancing and processing are some of the advantages and reason for preference of digital bitewing radiography technique [64]. In the research papers, there were numbers of study utilizing digital bitewing radiography [19, 20, 65–69]. It is a more beneficial technique than other x-ray containing methods for the simultaneous evaluation of the crowns and alveolar bone levels of posterior teeth in both upper and lower dental arch, caries detection that especially in approximal caries, and teeth detection and numbering [10, 70, 71].

**Fig. 11:**
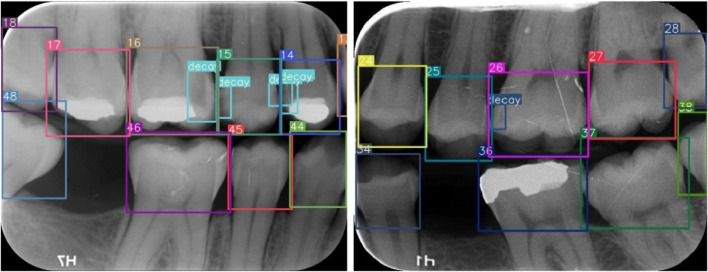
Sample of correct teeth numbering in cases where a tooth was missing

Also, there have been studies of the implementation of CNN to bitewing, panoramic, periapical, cone beam computed tomography radiographs and various diagnostic methods [34, 72, 73]. Zhang et al. [73] applied a DL method based on CNN for teeth detection and classification in periapical radiographs. Both precision and recall scores were high and, 95.8% and 96.1%, respectively. In our study, the improved model achieved recall of 99.4% and precision of 98.75%, in digital bitewing radiographs. In another study on CBCT radiographs reported that implementing a 3D U-Net network model showed satisfactory results for the segmentation and classification of teeth. Furthermore, an AI-based segmentation of all teeth in a single scan resulted in a speed improvement of 1800 times compared to that of an expert [72]. Tuzoff et al. [34] utilized the Faster R-CNN model for tooth detection and the VGG-16 Net model for tooth numbering on panoramic radiographs. The CNN-based models demonstrated high recall and specificity values for teeth detection and numbering tasks, comparable to experts. However, their model exhibited misclassifications in cases where a tooth was missing, whereas our improved model accurately detected these cases. Image samples for these cases are given in Fig. 11.

A DL method Faster R-CNN proposed for tooth detection and numbering in periapical radiographs by Chen et al. [57]. In order to improve the detection accuracy, they proposed three post-processing techniques to complement the baseline Faster R-CNN. Their model achieved precision of 98.8% and recall of 98.5% for detection, and precision of 91.7% and recall of 91.4% for numbering. In their study, the model was unable to accurately predict teeth located at the corners of the bitewing image. However, our improved model demonstrated a precision of 98.75% and a recall of 99.4% for detection, as well as a precision of 98.5% and a recall of 97.4% for numbering.

In literature, researchers generally benefit image enhancement and cropping techniques to improve accuracy. Mao et al. [74] improved the new Alexnet model, a CNN-based approach for caries and restoration detection on bitewing images, by utilizing image enhancement and cropping techniques. The model showed accuracy of 90.30% for caries detection and 95.56% for restoration detection. However, it is important to note that high accuracy scores alone may not indicate the reliability of a method. If a CNN model labels every surface as "decayed," it can achieve perfect accuracy but lack meaningful results. To address this, recall and specificity scores are reported in our study, following the precedent set by previous research, to provide a more comprehensive evaluation without sacrificing interpretability. Besides, we did not use any image enhancement or cropping methods.

In a previous study, an automatic assistance system developed for detecting caries lesions in bitewing radiographs using a U-Net model. The system's performance was evaluated through a comparative analysis with dentists. According to the findings of this study, the model demonstrated an overall accuracy of 80%, while the mean accuracy of practitioners was 71%. The recall, specificity, and F1-score values were also high, at 75%, 83%, and 73%, respectively [20]. Although the results of the model in this study were not directly compared with the performance of human experts, it showed promising results in the detection of caries lesions, achieving recall, precision and F1 score values of 83.3%, 86.6% and 82.2%, respectively. These results are very similar to those reported in the study by Cantu et al. In addition, the present study evaluated the performance of tooth numbering simultaneously with the detection of caries lesions.

Yasa et al. [29] proposed a Faster R-CNN model for the detection and numbering of teeth in bitewing radiographs. They conducted a comparison between two different models for tooth numbering. Initially, the teeth were not separated into right and left sides. However, after splitting and training the model in this manner, promising results were obtained, with recall, precision, and F1-score values of 97.48%, 92.93%, and 95.15%, respectively. In our study, we adopted the approach of separating teeth into right and left sides, following a similar methodology. Through the adoption of this approach, our improved model exhibited promising performance, as indicated by notable recall, precision, and F1-score values of 97.4%, 98.5%, and 97.9%, respectively.

In another study, Bayraktar and Ayan [19] reported detection of interproximal caries lesions in digital bitewing radiographs with YOLOv3 model. In that study, the recall was 72.26%, precision was 86.58%. According to these promising results, the use of CNN-based system could be useful in diagnosing caries lesions. In the current study, our improved model is YOLOv7-AP-CBAM model with implementation of CBAM module. Through to CBAM module, the model could focus more precisely. In this way, the model reached recall of 83.3% which is higher than the previous study. However, the precision values were nearly same which are 86.58% and 86.6%.

The present study, YOLOv7-AP-CBAM model exhibited promising results in terms of the evaluated parameters. In the confusion matrix that we created, while the specificity score is higher, contrary effect was noted in the recall score. This result showed that the improved model can accurately predict tooth numbering while also detecting sound surfaces, similar to the study by Lee et al. [22]. The overall accuracy, recall, specificity, precision and F1-score values were 0.934, 0.834, 0.961, 0.851 and 0.842, respectively.

The performance of a CNN model is directly dependent on the amount of training data. The use of large and diverse data sets in the training process improves the consistency and accuracy of the CNN models. Therefore, the quality and quantity of training data play a vital role in ensuring the effectiveness of CNNs [75]. In this study, training dataset was relatively small. Although the training dataset contains an equal number of right and left bitewing images, the model was more successful on the left side in detecting caries in the final results. This can be explained by the higher amount of caries in the bitewing radiographs on the left side in the training group. To avoid this problem, new bitewing images including caries may be added to the right-side training set. Dataset bias is another problem that occurs when specific samples in a dataset are over or under-represented. In order to avoid this problem, the dataset was generated images with taken from the same devices in various clinics with different resolutions and exposure times. However, the images obtained from different x-ray machines and from people with different demographic characteristics may affect the results. The proposed model's parameters were determined using validation data during the training phase, and the model's performance on unseen data was evaluated using different test data from the training and validation data. CNN models require high hardware requirements, especially in the training phase. In our study, we performed our training and tests on 1080 Ti graphic card. Computational performance will be higher on more advanced graphics cards. The dental caries lesions that were not evaluated using the gold standard method of histological assessment detected and labeled by the annotators on the radiographic images [76]. The model encountered challenges in accurately predicting teeth positioned at the corners of the bitewing image, as opposed to other teeth. This could be related to the reduced clarity of these specific teeth and the limited amount of data in the training set. Also, the improved YOLOv7-AP-CBAM model was not compared with an expert. Despite the limitations of the study, it is worth noting that there are several significant strengths present as well. The present study offered a comprehensive analysis by integrating the results of tooth detection, numbering, and caries detection. This comprehensive approach facilitated a more cohesive comprehension of the outcomes. Additionally, the experimental design included the split of data into right and left sides, enabling a more detailed analysis. This approach enhanced the performance metrics of our results. Furthermore, by adopting an interdisciplinary approach, our study introduced a novel perspective and makes an innovative contribution to previous papers.

## Conclusion

In this study, an improved YOLOv7-AP-CBAM model was proposed for accurate teeth detection, numbering and caries detection, concurrently assessing with which tooth had caries by matching the numbered teeth and the detected caries. For teeth numbering and caries detection matching performance; the accuracy, recall, specificity, precision and F1—Score values 0.934, 0.834, 0.961, 0.851 and 0.842, respectively. The model exhibited high recall, specificity, precision, and F1-score, highlighting the potential of CNNs. This result revealed that CNNs can provide valuable support to clinicians by automating the detection and numbering of teeth, as well as detection of caries on bitewing radiographs. This can potentially contribute for assessment, improve overall performance, and ultimately save precious time.
